# Fibrinogen-like protein 1 promotes liver-resident memory T-cell exhaustion in hepatocellular carcinoma

**DOI:** 10.3389/fimmu.2023.1112672

**Published:** 2023-03-13

**Authors:** Changjie Yang, Qiwei Qian, Yudong Zhao, Bingyuan Huang, Ruilin Chen, Qiyu Gong, Hao Ji, Chenchen Wang, Lei Xia, Zhengrui You, Jianjun Zhang, Xiaosong Chen

**Affiliations:** ^1^ Department of Liver Surgery, Ren Ji Hospital, Shanghai Jiao Tong University, Shanghai, China; ^2^ Division of Gastroenterology and Hepatology, Ren Ji Hospital, Shanghai Jiao Tong University, Shanghai, China; ^3^ Shanghai Institute of Immunology, Shanghai Jiao Tong University, Shanghai, China

**Keywords:** hepatocellular carcinoma, tissue-resident memory T cells, lymphocyte activation gene 3, exhaustion, granzyme B

## Abstract

**Background and aims:**

The key role of tissue-resident memory T (T_RM_) cells in the immune regulation of hepatocellular carcinoma (HCC) has been investigated and reported, but the regulatory mechanism of tumor microenvironment on T_RM_ cells is still unclear. Lymphocyte activating gene 3 (LAG-3) is a promising next-generation immune checkpoint that is continuously expressed due to persistent antigen exposure in the tumor microenvironment. Fibrinogen-like protein 1 (FGL1) is a classical ligand of LAG-3 and can promote T cell exhaustion in tumors. Here, we excavated the effect of FGL1-LAG3 regulatory axis on T_RM_ cells in HCC.

**Methods:**

The function and phenotype of intrahepatic CD8^+^ T_RM_ cells in 35 HCC patients were analyzed using multicolor flow cytometry. Using a tissue microarray of 80 HCC patients, we performed the prognosis analysis. Moreover, we investigated the suppressive effect of FGL1 on CD8^+^ T_RM_ cells both in *in vitro* induction model and *in vivo* orthotopic HCC mouse model.

**Results:**

There was an increase in LAG3 expression in CD8^+^ T_RM_ cells in end-stage HCC; moreover, FGL1 levels were negatively correlated with CD103 expression and related to poor outcomes in HCC. Patients with high CD8^+^ T_RM_ cell proportions have better outcomes, and FGL1-LAG3 binding could lead to the exhaustion of CD8^+^ T_RM_ cells in tumors, indicating its potential as a target for immune checkpoint therapy of HCC. Increased FGL1 expression in HCC may result in CD8^+^ T_RM_ cell exhaustion, causing tumor immune escape.

**Conclusions:**

We identified CD8^+^T_RM_ cells as a potential immunotherapeutic target and reported the effect of FGL1-LAG3 binding on CD8^+^ T_RM_ cell function in HCC.

## Introduction

Hepatocellular carcinoma (HCC) is an increasing global concern, ranking sixth in incidence and third in mortality among cancers (1). Immune checkpoint blocking (ICB) therapy, which focuses on reactivating the normal functions of T-cells, is an emerging treatment for liver cancer (2). However, while anti-PD-1/anti-PD-L1 and anti-CTLA-4 monoclonal antibodies (mAbs) have shown potential in eliminating tumor growth, the long-term survival remains poor (3–5). Therefore, it is necessary to identify other immune checkpoint pathways by exploring the immune escape mechanism in depth.

T_RM_ cells are a group of non-circulating CD8^+^T cells that express CD69 and CD103 surface markers and permanently reside in peripheral tissues, where they readily mediate regional tumor immune surveillance (6–8). CD8^+^ T_RM_ cells are involved in many liver diseases and are necessary for the pathogeneses of primary biliary cholangitis and autoimmune hepatitis (9). T_RM_ cells are well-known for their immune reactions against infectious diseases, and there is growing evidence that they are pivotal in suppressing solid cancer growth (10–12). T_RM_ cells secrete cytokines to mediate CD103-enhanced tumor cell killing and maintain tumor immune surveillance (6). The dynamics of CD103^+^ T_RM_ and exhausted T cells also affect patient outcomes in HCC (13). Recently, T_RM_ cells were shown to be the first responders upon pre-surgical ICB therapy in oral squamous cell carcinoma (14). Therefore, in order to improve HCC immunotherapy, targeting CD8^+^ T_RM_ cells may be a viable approach.

Lymphocyte activation gene 3 (LAG3), a transmembrane protein found predominantly in activated T cells, regulates CD8^+^ T-cell proliferation, activation, effector functions, and homeostasis (15–17). LAG3 is a marker of CD8^+^ T-cell exhaustion, which occurs in response to repetitive antigenic stimulation in chronic viral infections and cancer (18–20). Several clinical trials have evaluated the anti-tumor activity of mAbs that block the interaction between LAG3 and its classical ligands, the MHC-II molecules (19, 20). Further, T_RM_ cells exhibit elevated LAG3 expression during viral infections; and blocking the LAG3 pathway can restore the antiviral response and improve T_RM_ cell functions (21). Fibrinogen-like protein 1 (FGL1), an emerging hepatic factor in the fibrinogen super-family, is mainly expressed in the liver under steady-state conditions and is abundantly expressed in hepatocellular carcinoma (22, 23). Chen et al. demonstrated that FGL1 could bind to LAG3 to suppress antigen-induced T-cell responses (24). However, the mechanism through which FGL1-LAG3 binding regulates T_RM_ cell immunity in hepatocellular carcinoma remains unclear.

Here, we investigated the effect of FGL1-LAG3 binding on CD8^+^ T_RM_ cells in HCC and explore new immune checkpoint therapy for HCC patients.

## Materials and methods

### Tissue samples

Blood was collected from healthy human donors, while tumors and matched adjacent normal tissues were donated by patients with HCC who had undergone partial hepatectomy or orthotopic liver transplantation at Ren Ji Hospital (Shanghai Jiao Tong University, China). From June 2020 to July 2021, freshly harvested tissues (Cohort 1, n = 35) were obtained and preserved. A tumor microarray (TMA) was developed using HCC samples (Cohort 2, n = 80) obtained from May 2015 to April 2016. Relevant clinical information is shown in **Supplementary Tables 1**, **2**. Histological diagnoses were confirmed by two pathologists. All patients provided informed written consent before surgery. Protocols regarding the application of these samples were drafted and approved by the Ethics Committee of Ren Ji Hospital (permit number: KY2020–055).

### Intrahepatic lymphocyte isolation and PBMC isolation

Intrahepatic lymphocytes were segregated as previous studies (25). Liver samples were carved into small pieces, digested with 0.01% collagenase IV and tapped four times per s for 2 min using a tapping homogenizer. By gently grinding through a 70-µm cell strainer, the homogenate was segregated. After centrifuged, the cells were collected for flow cytometry. Human peripheral blood mononuclear cells (PBMCs) were isolated from fresh blood *via* gradient centrifugation using Ficoll–Hypaque Plus (GE Healthcare).

### *In vitro* generation of human CD8^+^ TRM cells

A previously reported CD69^+^CD103^+^CD8^+^ T_RM_ cell polarization assay was used *in vitro* (26). To further explore the influence of FGL1 on CD8^+^ T_RM_ cell proliferation, FGL1 (100 nmol/mL; R&D Systems) was supplemented to the culture medium of PBMCs pretreated with IL-15 and TGF-β. Cells were harvested and assessed using flow cytometry on the sixth day.

### IHC and confocal microscopy

According to the procedure described previously (27), formalin-fixed, paraffin-embedded liver tissues were used for immunohistochemistry (IHC) and immunofluorescence with the primary antibodies targeting at CD103, CD8, TGF-β, IL-15 (all anti-human, ab129202, ab17147, ab170874, ab55276, Abcam, Cambridge, United Kingdom), and FGL1 (anti-human, ab275091, Abcam; anti-mouse, 16000-1-AP, Proteintech, San Diego, CA, USA). Then information of antibodies could be found in the **Supplementary table**. For the quantification of IHC data in Cohort 1, five areas of each liver section were randomly selected and assessed by two independent observers. The expression of CD103^+^ were evaluated by calculating the number of each high-magnification field of view. The expression of FGL1 in cohort 1 was scored from 0 to 4 according to its staining density, which was averaged from five captured high-powered field. A tissue microarray (TMA) of 80 HCC tissues from cohort 2 was employed to assess the prognostic value of CD103 and FGL-1 according to the elaborated survival data. We quantified the expression of CD103 and FGL1 in each patient in TMA by measuring the number of positive cells per area (28). Confocal scans were performed using an LSM-710 laser scanning confocal microscope (Carl Zeiss, Germany).

### Database

Correlations of CD103 gene expression with the genes of immune checkpoints’ ligands in HCC were estimated *via* tumor immune estimation resource (TIMER)database. TIMER (https://cistrome.shinyapps.io/timer/) is based on TCGA data and can be used to systematically analyze the immune infiltrates of different cancer types online.

### Kaplan-Meier analysis

Survival and correlation analyses were performed based on the follow-up information from patients from cohort 2. The expression of CD103 and FGL1 was grouped into low-level, medium-level, and high-level categories according to the tri-sectional quantiles. Kaplan-Meier survival analysis (i.e., the product-limit method) was employed to assess the probability of survival based on the statistics of observed survival time. Let t(i) denote ordered event times (not counting censoring times), *d*(i) denote the number of events at *t*(i), and *n*(i) denote the total of alive cases, which is hence at risk before *t*(i). Survival probability at t is calculated using the equation below.


$$\hat{S}t=\prod_{i:t_{i}\leq t}\left(1-\frac{d_{i}}{n_{i}}\right)$$

The R package survival and survminer were taken for KM analysis and visualization.

### Cox proportional hazards model

To relate survival times and predictors, including categorical variables, such as sex, and other continuous variables, such as age, the Cox proportional hazards model was used, which does not assume the shape of the underlying hazard. Let j(i) denote a person failing at t(i) and let R(i) denote the risk set of all persons alive just before t(i). exp(xβ) indicates the relative risk for a subject with covariate values compared to a subject at baseline. The probability that the person who failed at *t*(i) would be *j*(i) conditional on the risk set is calculated using:


$$L_{i}=\frac{e^{x_{j\left(i\right)}\text{β}}}{\sum_{\in R_{i}}e^{x_{j}\text{β}}}$$

The R package survival (v3.2) and forest plot (v2.0.1) were used for univariate Cox analysis and visualization. The ratio of positive cells was used to calculate the Spearman covariance between CD103 and FGL1 expression. The R package ggplot2 (v3.3.5) was used for visualization.

### Quantitative real-time PCR

A tissue total RNA isolation kit (Vazyme, RC112) was employed to extract total RNA from the liver tissues, while a reverse transcriptase kit (Vazyme, R323-01) was used to synthesize cDNA. RT-qPCR was conducted through the use of SYBR Premix Ex Taq (Takara) with an ABI PRISM 7900HT sequence detector. A housekeeping gene, *GAPDH*, was used as an endogenous control. The data were evaluated by employing the 2^-ΔΔCt^ method. Primer sequences could be found in the table of sequence-based reagents.

### Western blot

The WB was conducted as reported previously (29). Relative quantities of protein were determined by comparing to GAPDH expression and analyzed using ImageJ 1.46 r. The antibodies used were mouse anti-GAPDH (1:10,000, Abcam, ab8245), rabbit anti-TGF-β (1:1000, Abcam, ab170874), rabbit anti-IL-15 (1:1000, Abcam, ab55276), and rabbit anti-FGL1 (1:1000, Abcam, ab38007).

### Flow cytometric analysis

The flow cytometric was conducted as reported previously (27). For surface staining, the cells were cultured with antibodies specific for human cell surface markers CD3, CD4, CD8, CD103, CD69, LAG-3, PD-1, TIM-3, and CTLA-4 (564713, 564724,564116, 350225, 557745, 565775, 329920,

748820, 563931, all from BD Biosciences) for 30 min at 4°C. For intracellular cytokine staining (ICS), immune cells were first incubated in complete culture medium containing a leukocyte activation cocktail with GolgiPlug (BD Biosciences) at 37°C for 5 h. The cells were then stained with surface keratin and fixed with a BD Cytofix/Cytoperm kit for 20 min at 4°C. The cells were then stained with antibodies against IFN-γ, TNF-α (563416,559321, BD Biosciences), IL-2 and granzyme B (500310, 372221, Biolegend) for 30 min at 4°C. For nuclear factor staining, the cells were first stained with surface markers and then fixed with a transcription factor buffer set (BD Biosciences) for 50 min at 4°C. Next, the cells were stained with Runx3, T-bet (564814, 564141, BD Biosciences), TOX, and Eomes (50-6502-80, 25-4875-82, eBioscience) for 50 min at 4°C. All samples were detected using flow cytometry (BD LSR Fortessa, BD LSR Fortessa X20) and analyzed using FlowJo (v10.6.2, Tree Star, Ashland, USA).

### Cell culture and reagents

Hepa1-6 cells were acquired from the Type Culture Collection of the Chinese Academy of Sciences (Shanghai, China). According to manufacturer’s instructions, we transfected two short hairpin RNAs (shRNAs) targeting the *Fgl1* coding sequence to inhibit the expression of FGL1, which were purchased from Shanghai Zorin Biotechnology. The cells were cultured for 5 days, and then mRNA was quantified by Western blot. The shRNA sequences could be found in the table of sequence-based reagents.

### Colony formation assays

Hepa1-6 and sh-*Fgl1* Hepa1-6 cells were digested using trypsin, terminated in culture medium, and resuspended after centrifugation. Cell suspensions were prepared as concentration gradient dilutions, and each group of cells was added to 6-well plates at a concentration of 20, 40, and 80 cells per well. The medium was replenished (2 ml), and the cells were incubated in the medium for two weeks. The supernatant was discarded when visible clones appeared in the culture dish. After washed three times by PBS, the cells were fixed with an appropriate amount of 4% paraformaldehyde. An appropriate amount of Gimusa staining solution was added to the cells for half an hour. The 6-well plates were then rinsed with running water. After drying, photographs of the plates were obtained. Image analysis was performed using ImageJ (v1.46)

### Animal studies

Twenty male C57BL/6J mice, 6 weeks old (SIMM Animal Center, Shanghai, China), specific pathogen-free, were administered a standard diet in the laboratory and provided with sufficient water, with their accommodation conditions in accordance with laboratory standards. All experiments of mice strictly followed the ethical guidance regarding animal care and were officially approved by the Institutional Animal Care and Use Committee at the Shanghai Institute of Materia Medica (approval no. 2021-08-LC-26). An orthotopic HCC murine model was established based on a previous study (30). The mice were randomly divided into four groups of five: untreated, sham operation, control, and sh-*Fgl1*. After anesthesia with isoflurane, the mice were injected in the left hepatic lobe with PBS (referred to as the sham-operation group), 5 × 10^6^ wild-type (WT) Hepa1-6 tumor cells (referred to as the control group), or 5 × 10^6^ *Fgl1-KO* Hepa1-6 tumor cells (referred to as the sh-*Fgl1* group). After 14 days, the mice were euthanized, while the tumor sizes were measured based on width and length using vernier calipers. The corresponding volumes were calculated *via* the equation as follows: Volume=length*(width)^2^*π/6. To estimate the potential influences of FGL1 on CD8^+^ T_RM_ cells with respect to survival rates in HCC mice, another set of mice was used as previously described (10 animals/group). The survival of the mice was assessed daily. And the survival curve was plotted by using GraphPad (v8.0).

### Statistical analyses

All statistical tests were conducted through the use of GraphPad (v8.0) and R software (v4.2.1). Statistical differences in averagely distributed data were studied using the Student’s *t*-test. A paired *t*-test was conducted to contrast the number of CD8^+^T_RM_ cells between HCC and adjacent tissues. The survival time was accumulated using the Kaplan–Meier method and further examinzed using the log-rank test. The expression scatterplots of correlations were studied using Spearman’s rank correlation. The ratio of positive cells was used to calculate the Spearman covariance between CD103 and FGL1 expression. *P< 0.05 was regarded essential.

## Results

### Increased CD103 expression in tumors is correlated with a better prognosis for HCC patients

**Figure 1:**
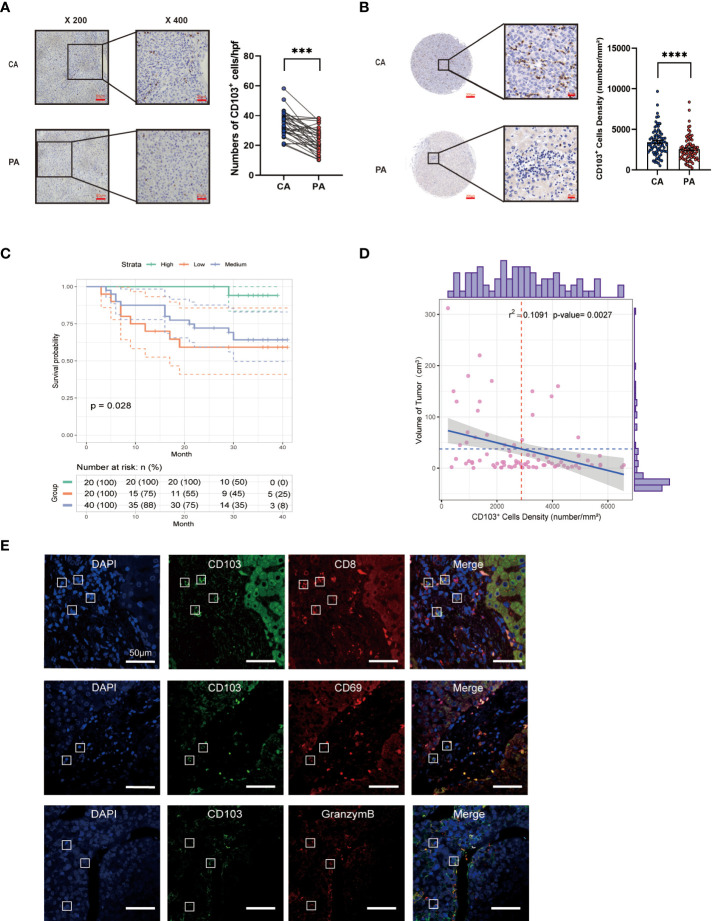
Hepatic CD69^+^CD103^+^CD8^+^ T-cell number is increased in HCC and is correlated with good prognosis. **(A)** Representative IHC staining of CD103 (inverted microscope, 200×, 400×) in CA and PA. Quantification of CD103 in the HCC patients from Cohort 1 (n = 35, A paired *t*-test). **(B)** Representative IHC staining of CD103 (inverted microscope, 200×, 400×) in TMA. Quantification of CD103 in the HCC patients from Cohort 2 (n = 80, A paired *t*-test). **(C)** Kaplan–Meier curves for the overall survival of the Cohort 2 groups with high, medium, and low CD103 expression, according to tri-sectional quantiles, showed that the patients with high CD103 expression had better prognostic outcomes than those with low CD103 expression (n = 80, log-rank test*, P =* 0.028). **(D)** Correlation between CD103^+^ cell density and tumor volume (n = 80, Spearman r^2^ = 0.1246, *P* = 0.001321) **(E)** Representative confocal assay of CD103 with CD69, CD8, and GZMB (400×) in the liver tissues of HCC patients from Cohort 1 (n = 8). **(A–E)** Bars represent mean ± SEM. CA, carcinoma tissue; GZMB, granzyme B; HCC, hepatocellular carcinoma; IHC, immunohistochemistry; PA, paracancerous tissue; TMA, tumor microarray. ***P< 0.001, ****P< 0.0001.

First, IHC staining for CD103 in the HCC tissues from Cohort 1 revealed significantly more CD103^+^ cells in HCC (*P<* 0.001, **Figure 1**). To reveal the correlation between the number of CD103^+^ cells in liver cancer tissues and patient outcomes, IHC staining of CD103 was then performed using the TMA, which was collected from 80 patients in Cohort 2 (*P<* 0.001). By further using a TMA containing 80 HCC patients, we not only confirmed a higher number of CD103^+^ cells in cancer, but also found a positive correlation between the number of CD103^+^ cells and better prognoses (*P* = 0.028). Moreover, the density of CD103^+^ cells was negatively correlated with tumor size in Cohort 2 (Spearman r^2^ = 0.1091, *P* = 0.0027, **Figures 1B, C**). Double immunofluorescence staining confirmed that the majority of CD103^+^ cells in the livers of HCC patients were CD69^+^CD8^+^. Confocal microscopy demonstrated that CD103^+^ lymphocytes in HCC produced granzyme B (GZMB) to maintain cytotoxicity (**Figure 1**). These findings confirmed that patients with more CD103^+^ cells in HCC have better prognoses, indicating that CD8^+^ T_RM_ cells in HCC are vital for anti-tumor immunity.

### Increased intrahepatic CD8^+^ TRM cells demonstrate exhausted phenotypes in HCC

**Figure 2:**
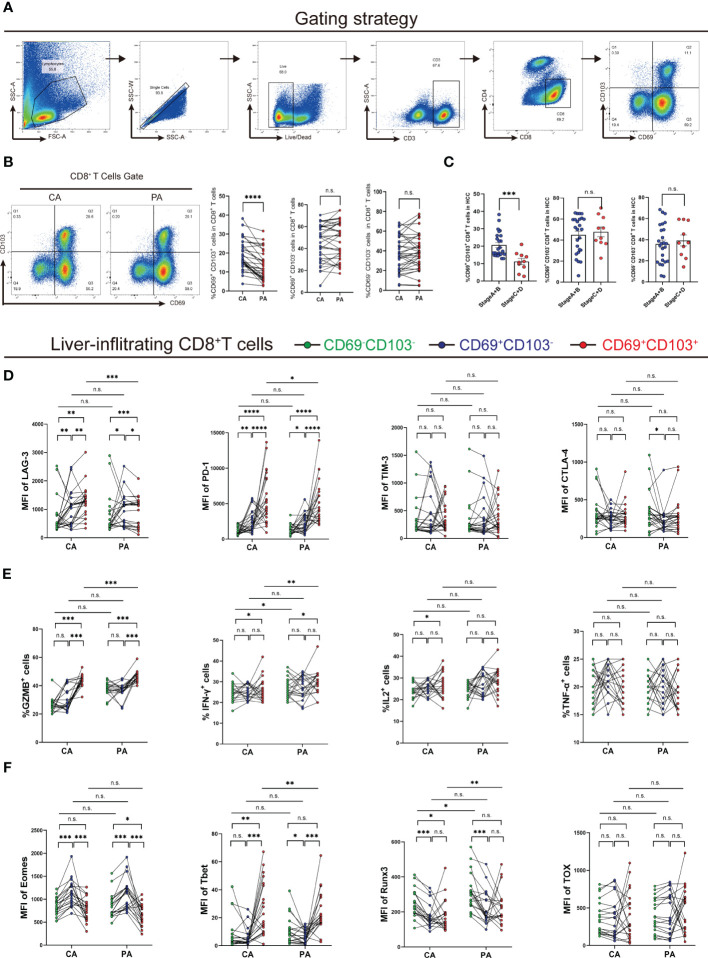
Analysis of the frequency, phenotype, and function of intrahepatic CD8^+^ T_RM_ cells. **(A)** Gating strategy of intrahepatic CD8^+^ T_RM_ cells. **(B)** Proportion of CD8^+^ T_RM_ cells and other CD8^+^ T-cell subpopulations in CA and PA in Cohort 1 patients (n = 35, A paired *t*-test). **(C)** Ratio of CD8^+^ T_RM_ cells and other CD8^+^ T-cell subpopulations in CA of Cohort 1 patients, which were grouped by the BCLC score (n = 35, Student’s *t*-test.). **(D)** Immune checkpoint expression on CD8^+^ T_RM_ cells and other CD8^+^ T-cell subpopulations in CA and PA of HCC patients from Cohort 1 (n = 20, A paired *t*-test). **(E)** Cytokine expression in CD8^+^ T_RM_ cells and other CD8^+^ T-cell subpopulations in CA and PA of HCC patients from Cohort 1 (n = 20, A paired *t*-test). **(F)** Transcription factor expression on CD8^+^ T_RM_ cells and other CD8^+^ T-cell subpopulations in CA and PA of HCC patients from Cohort 1 (n = 20, A paired *t*-test). **(B–F)** BCLC, Barcelona Clinic Liver Cancer; CA, carcinoma tissue; HCC, hepatocellular carcinoma; MFI, mean fluorescence intensity; PA, paracancerous tissue. n.s. means not siginificant, *P< 0.05, **P< 0.01, ***P< 0.001, ****P< 0.0001.

CD8^+^ T_RM_ cells from Cohort 1 liver tissues were assessed in terms of the proportion, phenotype, and function using flow cytometry (**Figure 2**). We observed a significantly higher frequency of CD8^+^ T_RM_ cells in cancer tissues than in paracancerous tissues (*P<* 0.0001, **Figure 2**). We then scored Cohort 1 patients based on Barcelona Clinic Liver Cancer (BCLC) staging classification and divided them into early-stage (A+B) and late-stage (C+D) groups. We found a higher frequency of CD8^+^ T_RM_ cells in patients with early-stage disease than in those with advanced stage (*P<* 0.001, **Figure 2C**).

Additionally, we detected the expression of immune checkpoints in CD8^+^ T-cell subsets, such as LAG3, PD-1, TIM-3, and CTLA-4. Expression levels of LAG3 and PD-1 in CD8^+^ T_RM_ cells were substantially higher than those in other CD8^+^ T-cell subpopulations in HCC tissues. Moreover, expression levels of LAG3 and PD-1 in CD8^+^ T_RM_ cells in HCC tissues were higher (**Figures 2D**, **S1E**). Next, we examined the levels of cytokines released by CD8^+^ T_RM_ cells after activation and found that intratumoral CD8^+^ T_RM_ cells released more GZMB and IFN-γ than other CD8^+^ T-cell subpopulations. Meanwhile, CD8^+^ T_RM_ cells released less GZMB and IFN-γ in HCC tissues than in paracancerous tissues. We observed no significant difference in TNF-α and IL-2 expression among CD8^+^ T-cell subpopulations (**Figures 2E**, **S1F**). We also examined transcription factors in the CD8^+^ T-cell subpopulations and discovered that eomesodermin (Eomes) was less expressed in CD8^+^ T_RM_ cells than in the two other groups of cells in HCC, while T-bet was more expressed in CD8^+^ T_RM_ cells. They had higher expression of Runx3 in HCC tissues than those in paracancerous tissues, and there was no difference in TOX expression (**Figure 2F**, **S1G**). These results showed that the percentage of intratumoral CD8^+^ T_RM_ cells had added, and their function changed.

### FGL1 negatively correlates with signature genes of CD8+ TRM cells in HCC *in silico*

**Figure 3:**
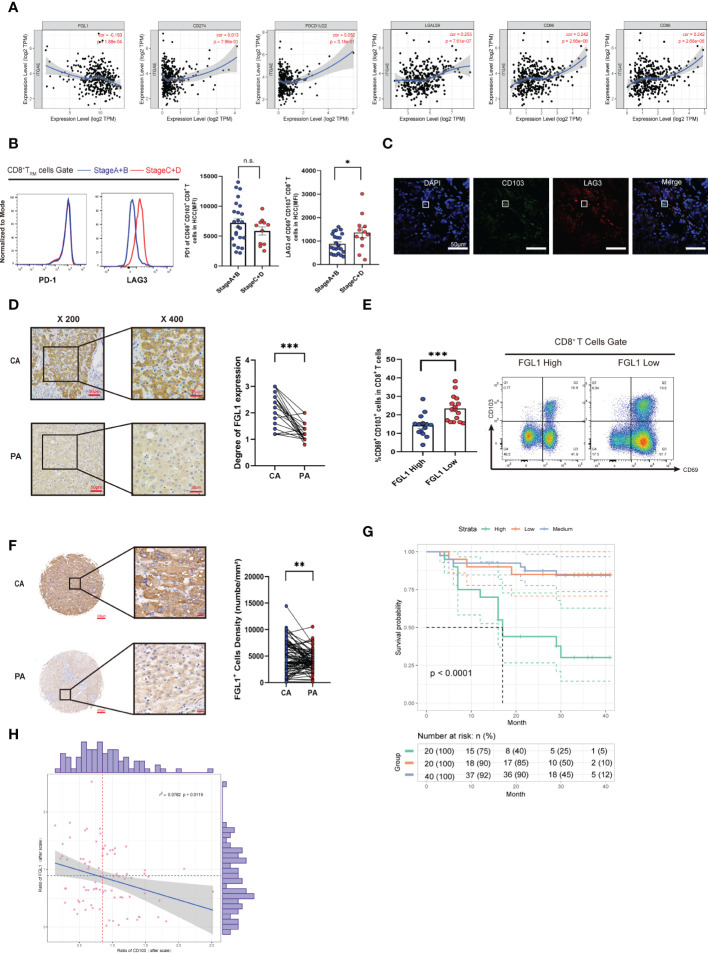
FGL1 expression is increased in HCC and correlates with poor prognosis. **(A)** Expression scatterplots of correlations between the genes of CD103 and FGL1, MHC-II, LSECtin, galectin-3, PD-L1, and PD-L2 in HCC of the TIMER cohort. Spearman correlation coefficient test was calculated. **(B)** MFI of PD-1 and LAG3 expression by CD8^+^ T_RM_ cells in the liver cancer tissues of Cohort 1 patients, which were grouped by the BCLC score (n = 35, Student’s *t*-test). **(C)** Representative confocal assay of CD103 with LAG3 (400×) in the liver tissues of HCC patients from Cohort 1 (n = 10). **(D)** Representative IHC staining of FGL1 (inverted microscope, 200×, 400×) in CA and PA of patients from Cohort 1 (n = 35, A paired *t*-test). **(E)** The frequency of CD103^+^CD69^+^CD8^+^ T cells in groups with high or low FGL1 expression in HCC patients from Cohort 1. The cutoff for FGL1-based grouping was determined by the median (n = 30, Student’s *t*-test). **(F)** Representative IHC staining of FGL1 (inverted microscope, 200×, 400×) in TMA. Quantification of FGL1 in the HCC patients from Cohort 2 (n = 80, Student’s *t*-test). **(G)** Kaplan–Meier curves for the overall survival of the groups with high, medium, and low FGL1 expression in Cohort 2, according to the tri-sectional quantiles, showed that the patients with high FGL1 expression had worse outcomes than those with low FGL1 expression (n = 80, *P<* 0.0001). **(H)** Correlation between CD103^+^ cell density and tumor volume (n = 80, Spearman r^2^ = 0.0755, *P* = 0.007). **(A–H)** Bars represent mean ± SEM. BCLC, Barcelona Clinic Liver Cancer; CA, carcinoma tissue; FGL1, fibrinogen-like protein 1; HCC, hepatocellular carcinoma; IHC; immunohistochemistry; MFI, mean fluorescence intensity; PA, paracancerous tissue. *P< 0.05, **P< 0.01, ***P< 0.001. n.s. means not siginificant.

We next questioned how the immune microenvironment of HCC regulates the phenotypes and functions of CD8^+^ T_RM_ cells. We first confirmed that key proteins associated with the T-cell residency phenotype, including IL-15 and TGF-β, were upregulated in HCC tissues using histological methods (**Figures S1A–S1D**), indicating that the HCC microenvironment is conducive to the development and residency of intrahepatic CD8^+^ T_RM_ cells. Despite the increased number of intrahepatic CD8^+^ T_RM_ cells, their function was impaired. Expression of immune checkpoint ligands (ICLs) is necessary to trigger inhibitory signaling *via* immune checkpoint receptors (ICRs) in exhausted T cells in the tumor immune microenvironment (31, 32). We then examined the correlations between the individual ICLs genes and *ITGAE* in the TIMER database and found that only FGL1, the ligand of LAG-3, showed a significant negative correlation with *ITGAE*, while other ICLs showed weak positive correlations (Spearman r = -0.19, *P* = 0.00018; **Figure 3A**) (33, 34). These data suggest that among different ICLs, FGL1 is the most important negative regulator of T_RM_ cells.

### Increased FGL1 in HCC is related to a poor prognosis

We found that the CD8^+^ T_RM_ cell surface in end-stage HCC expressed higher levels of LAG3 (n = 35, *P<* 0.05; **Figure 3B**), while there was no difference in the levels of PD-1. To further clarify the relationship between LAG3 and CD8^+^ T_RM_ cells, we performed confocal staining for LAG3 and CD103 expression in cells (**Figure 3C**). The colocalization of CD103 with LAG3 on the cell membrane provided a basis to explore how FGL1 affects intrahepatic CD8^+^ T_RM_ cells. We next explored the expression of FGL1 in HCC. As shown in **Figure 3**, FGL1 was expressed extensively in HCC (*P<* 0.001). Then, we separated patients into two groups by median based on their FGL1 level. As a result, patients with high FGL1 levels had a lower percentage of CD8^+^ T_RM_ cells. Thus, we speculated that FGL1 might restrict the population of CD8^+^ T_RM_ cells (**Figure 3E**). To further investigate whether FGL1 in liver cancer tissues is correlated with patient outcomes, we performed IHC staining for FGL1 using the TMA from Cohort 2 patients (*P<* 0.001, **Figure 3F**). The prognoses of patients with higher FGL1 levels were worse than those of patients with lower levels (*P<* 0.0001, **Figure 3G**). The levels of FGL1 correlated negatively with those of CD103 in Cohort 2(Spearman r^2^ = 0.0782, *P* = 0.0119, **Figure 3H**), which was consistent with the gene expression of the TIMER cohort. Univariate Cox regression analysis confirmed that FGL1 expression is an independent risk factor for HCC (**Table 1**). Thus, FGL1 may serve as an independent biomarker for the prognosis of patients with liver cancer.

**Table 1:** Univariate Cox regression analysis of potential prognostic factors in HCC.

Characteristics	HR (95%CI)			P−Value
AGE	-0.04	0.9607 (0.9184-1.0051)	3.03	0.0818
CD103	-0.0174	0.9828 (0.9669-0.9989)	0.0362	**0.0362**
FGL1	0.0296	1.03 (1.0125-1.0478)	11.41	**7e-04**
Glu	-0.0385	0.9623 (0.8681-1.0667)	0.54	0.4641
ALT	1e-04	1.0001 (0.9995-1.0007)	0.12	0.7338
TB	0.0065	1.0066 (1.0015-1.0116)	6.46	0.011
DB	0.008	1.008 (1.0005-1.0155)	4.37	0.0365
ALB	-0.0186	0.9816 (0.9368-1.0285)	0.61	0.4354
Hgb	-0.0071	0.9929 (0.9805-1.0055)	1.22	0.2687

HR, Hazard Ratio; CI, confidence interval; FGL1, fibrinogen-like protein 1; HCC, hepatocellular carcinoma; ALT, Alanine aminotransferase; TB, Total bilirubin; DB, Direct Bilirubin; Alb, Albumin; Hgb, hemoglobin. These bold values mean that CD103 and FGL1 are potential prognostic factors in HCC.

### FGL1 affects CD8^+^ T_RM_ cells *in vitro*

**Figure 4:**
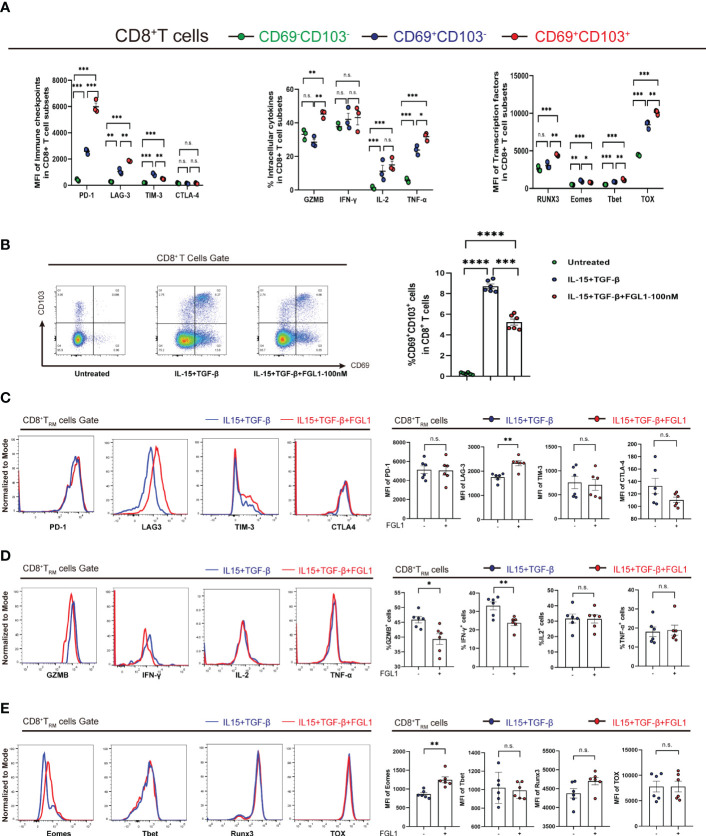
FGL1 expression inhibits the proliferation of CD8^+^ T_RM_ cells *in vitro* **(A)** PBMCs were cultured in the presence of IL-15 (50 ng/mL) for 3 days and then with TGF-β (50 ng/mL) for an additional 3 days to activate CD8^+^ T_RM_ cells. The expression of the markers of CD8^+^ T_RM_ cells and other CD8^+^ T-cell subpopulations was determined. **(B)** FGL1 (100 nM) was added to the cultures, and the percentage of CD8^+^ T_RM_ cells was detected after activation. **(C)** Expression of CD8^+^ T_RM_ cell surface molecules, such as PD-1, LAG3, TIM-3, and CTLA-4, was detected after activation. **(D)** Expression of CD8^+^ T_RM_ cell cytokines, such as IFN-γ, IL-2, and TNF-α, as well as GZMB, was detected after activation. **(E)** Expression of CD8^+^ T_RM_ cell transcription factors, such as Runx3, Eomes, T-bet, and TOX, was detected after activation. **(A–E)** A paired *t*-test. Bars represent mean ± SEM. FGL1, fibrinogen-like protein 1; GZMB, granzyme B; PBMC, peripheral blood mononuclear cell. (*P< 0.05, **P< 0.01, ***P< 0.001, ****P< 0.0001). n.s. means not siginificant.

To further investigate CD8^+^ T_RM_ cells in terms of their immune functions, a cellular model of CD8^+^ T_RM_ cells was generated *in vitro* as previously described (26). Briefly, PBMCs were cultured in 24-well culture plates at 5×10^5^ cells/mL in complete medium in the presence of 20 IU/ml rhIL-2 and incubated with IL-15 (50 ng/ml) for three days, followed by further incubation with TGF-β (50 ng/ml) for three days. Cells were harvested on the sixth day. Activated CD8^+^ T_RM_ cells displayed higher expression of LAG3, PD-1, TOX, and T-bet than CD69^+^CD103^-^CD8^+^ and CD69^-^CD103^-^CD8^+^ T cells, consistent with the CD8^+^ T_RM_ cell phenotype reported previously. Activated CD8^+^ T_RM_ cells also had higher Runx3 levels, contrasting with the results showing transcription factor levels in T-cell subpopulations in HCC, and there was no significant difference in Runx3 levels among the three intrahepatic populations (**Figure 4A**). We then added FGL1 to the activation system. The percentage of CD8^+^ T_RM_ cells decreased after the addition of FGL1 compared with that of the control group (**Figure 4B**). Next, we examined surface molecules, cytokines, and immune checkpoints to investigate the inhibitory effects of FGL1 on CD8^+^ T_RM_ cells. We found that the addition of FGL1 upregulated LAG3 expression on CD8^+^ T_RM_ cell membranes (**Figure 4C**), decreased the release of IFN-γ and GZMB (**Figure 4D**), and upregulated levels of the transcription factor Eomes (**Figure 4E**). These results suggested that FGL1 affects the killing function of CD8^+^ T_RM_ cells by binding to LAG3.

### Knockdown of *FGL1* does not significantly alter the proliferation of the mouse tumor hepa1-6 cell line

We constructed a Hepa1-6 mouse tumor cell line using lentivirus transfection and verified that shRNA-mediated *Fgl1* knockdown was effective using WB (**Figure S2A**). Cell line proliferation was also examined using a clone formation assay. We found no significant change in the proliferation between *FGL1*-knockdown and control Hepa1-6 cell lines (**Figure S2B**). The results indicated that FGL1 did not directly affect the proliferation of tumor cells but possibly affected CD8^+^ T_RM_ cells *via* FGL1-LAG-3 binding. The exact mechanism requires further verification using animal experiments.

### Mice in the *Fgl1*-knockdown group have smaller tumors and improved prognosis

**Figure 5:**
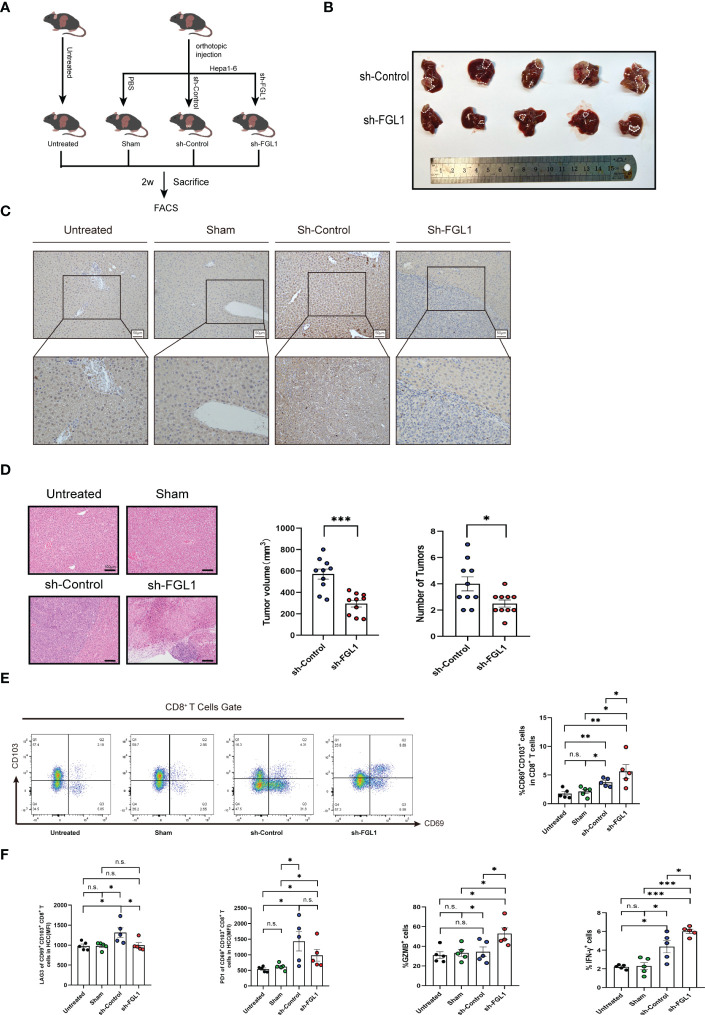
*Fgl1* knockout restored CD8^+^ T_RM_ cell functions and inhibited tumor expansion *in vivo* **(A)** C57BL/6J mice were classified into four groups and euthanized 14 days after orthotopic injection with PBS or Hepa1-6 cell line (transferred with sh-Control or sh-Fgl1, n=5). **(B)** Representative images of the tumors from the sh-*Fgl1* and sh-Control groups. **(C)** Representative IHC staining of FGL1 (inverted microscope, 200×, 400×) in the livers of mice in each group. **(D)** Representative H&E-stained sections of each group. **(E)** Percentage of CD8+ T_RM_ cells in each group. **(F)** Expression of the immune checkpoints PD-1 and LAG3 as well as GZMB and IFN-γ in CD8+ T_RM_ cells. **(A–F)** n >5, a paired t-test. Bars represent mean ± SEM. FGL1, fibrinogen-like protein 1; H-E, hematoxylin-eosin; IHC, immunohistochemistry; sh, short hairpin (RNA). n.s. means not siginificant. *P < 0.05, **P < 0.01, ***P < 0.001.

We used *Fgl1*-knockdown Hepa1-6 cell lines and control Hepa1-6 cell lines and constructed HCC models by inducing tumorigenesis *in situ*. Briefly, after anesthesia with isoflurane, the mice were injected in the left hepatic lobe with PBS (referred to as the sham-operation group), 5 × 10^6^ wildtype (WT) Hepa1-6 tumor cells (referred to as the control group), or 5 × 10^6^ *Fgl1*-KO Hepa1-6 tumor cells (referred to as the sh-*Fgl1* group). The experimental groups were as follows: untreated group; sham-operation group; control group (sh-control); and knockdown group (sh-*Fgl1*). The knockdown of *Fgl1* in Hepa1-6 cells inhibited tumor growth (**Figure 5A**). Heterotypic cells were found in the liver tissues of the control group in a nest-like arrangement with vacuolated nuclei and infiltrative growth. In the knockdown group, the size of the cancer nests was reduced, and the infiltrating area became smaller (**Figure 5B**). In the *Fgl1*-knockdown group, the expression of FGL1 in the mouse liver was substantially lower than that in the control group (**Figure 5C**). Further, the tumor volumes in mice were significantly reduced, and we observed a decrease in the number of cancer nodules (**Figure 5D**). The volumes were calculated *via* the equation as follows: Volume=length*(width)^2^*π/6. Mice treated as above mentioned were further used to estimate the influence of sh-*Fgl1* on overall survival. For this, the survival of mice was assessed daily. Differences were also observed in survival curves, with mice in the sh-*Fgl1* group having better survival. In the *Fgl1*-knockdown group, the serum biochemical parameters were reduced (**Figure S2C**).

### Mice in *Fgl1*-knockdown group have more CD8^+^ T_RM_ cells and lower LAG3 expression in HCC

To discover the regulatory effect of FGL1 on CD8^+^ T_RM_ cells *in vivo*, we euthanized mice from four groups 2 weeks after orthotopic injection, obtained cancerous tissues from the control and *Fgl1*-knockdown groups, and extracted non-parenchymal cells from the liver for flow cytometric analysis. The results demonstrated no difference in the percentage of CD8^+^ T_RM_ cells in the sham-operation and untreated groups, but increases in the control and knockdown groups, which is consistent with our previous finding of an increased proportion of CD8^+^ T_RM_ cells in human HCC liver tissues. The increased number of CD8^+^ T_RM_ cells in the *Fgl1*-knockdown group suggests that FGL1 affects CD8^+^ T_RM_ cells by binding to LAG3, causing a decrease in the number of exhausted CD8^+^ T_RM_ cells (which lead to immune escape) (**Figure 5E**). The *Fgl1*-knockdown group showed lower LAG3 expression but released more IFN-γ cytokines and GZMB than the control group (**Figure 5F**).

## Discussion

With ICB therapy recognized as a robust clinical strategy for the pre-surgical treatment of cancers, a better understanding of early responder T_RM_ cells is needed (14). For the optimal application of ICBs and the design of novel immunotherapeutic strategies for HCC patients, it is crucial to understand the tumor immune microenvironment. Herein, we discovered that most CD69^+^CD103^+^CD8^+^ T_RM_ cells are terminally differentiated in HCC and can secrete GZMB. This unique immune microenvironment was found to contribute to T_RM_ differentiation and accumulation in HCC. Additionally, we show that T_RM_ cells residing in HCC highly express LAG3 and PD-1, both of which indicate T_RM_ cell exhaustion. Furthermore, we found that FGL1, a primary ligand of LAG-3, is highly expressed in HCC. According to *in vitro* and *in vivo* experiments, FGL1 leads to T_RM_ suppression in HCC, and blockade of the LAG3 and FGL1 pathways might restore the T_RM_-mediated anti-tumor response in liver cancer. These results suggest that T_RM_ cells have anti-tumor potential for liver cancer and that FGL1-LAG3 binding could become a potential immune checkpoint for HCC therapy.

To study the specific immunological characteristics of CD8^+^ T_RM_ cells, we compared CD69^+^CD103^+^CD8^+^ T cells with other CD8^+^ counterparts, which are more abundantly distributed in the liver, in terms of the levels of surface molecules, cytokines, and transcription factors. We found that CD8^+^ T_RM_ cells expressed higher levels of PD-1 and LAG3 and lower levels of Eomes, IFN-γ, and GZMB in HCC than in paracancerous tissues. CD8^+^ T_RM_ cells in HCC display unique immunological characteristics compared with other CD8^+^ T-cell subpopulations, such as high expression of immunosuppressive molecules. According to these findings, CD69^+^CD103^+^ T cells consist of heterogeneous populations, which retain a proportion of cells that have the potential to be reinvigorated and to further proliferate in response to therapeutic interventions.

LAG3, a marker of CD8^+^ T-cell exhaustion, is mainly expressed on the membrane of activated T cells (35). LAG3 was found to colocalize with CD8^+^ T_RM_ cells in HCC. But the expression of its ligand FGL1 in hepatocellular liver cancer is controversial: a study in 2022 at Zhejiang Provincial People’s Hospital showed low expression of FGL1 in hepatocellular carcinoma, but high expression of FGL1 in hepatocellular carcinoma was reported in 2020 at Zhongshan Hospital (36), and in the same year a study from Youjiang Medical University for Nationalities reported that oxytocin could inhibit the growth of hepatocellular carcinoma by reducing FGL1 and sensitizing anti-LAG3 treatment and inhibiting hepatocellular carcinoma growth (37). We investigated the clinical relevance of FGL1 in HCC tissues using TMA IHC staining. Correlation analysis demonstrated that the proportion of FGL1^+^ cells negatively correlated with that of CD103^+^ cells in HCC. Thus, we hypothesized that FGL1 inhibits the expansion of CD8^+^ T_RM_ cells in this disease.

In functional studies of liver CD8^+^ T_RM_ cells, IL-15 and TGF-β have been sequentially used to activate CD8^+^ T_RM_ cells *in vitro* (26). The activated CD69^+^CD103^+^CD8^+^ T_RM_ cells had higher LAG3, PD-1, and Runx3 expression and reduced Eomes expression and GZMB secretion. These results are in accordance with the results of the phenotype of CD8^+^ T_RM_ cells reported previously. These data confirm the feasibility of the activation protocol. We added FGL1 protein to the activation process of CD8^+^ T_RM_ cells and found that FGL1 reduced the number of CD8^+^ T_RM_ cells and GZMB secretion *in vitro*. Further, Eomes expression was upregulated under the influence of FGL1, while downregulation of the T-box family, including Eomes and T-bet combinations, is critical for TGF-β cytokine signaling (38). In addition, one study showed that Eomes suppresses the formation of precursor and mature T_RM_ cells (39).

*In vivo* experiments were further conducted to investigate the inhibitory effects of FGL1 on CD8^+^ T_RM_ cells. There was no significant change in the proliferation of liver cancer cell lines in the *Fgl1*-knockdown Hepa1-6 murine model compared with that in the sham group. However, the *Fgl1*-knockdown group showed a substantial reduction in tumor size, cancer nodules, and liver enzymes, as well as a longer survival time and more CD8^+^ T_RM_ cells in HCC. Furthermore, the level of LAG3 in CD8^+^ T_RM_ cells was lower in the *Fgl1*-knockdown group. FGL1 inhibited the proportion and function of CD8^+^ T_RM_ *in vivo*, correlated with poor prognosis in HCC.

Our study had several limitations. First, we did not successfully sort CD8^+^ T_RM_ cells using flow cytometry or isolate human primary tumor cells for co-culture; thus, further clarification of the anti-tumor function of CD8^+^ T_RM_ cells is needed. Second, further studies are required to determine whether the suppression of CD8^+^ T_RM_ cells by FGL1 could be antagonized by inhibition of Eomes; however, there is currently no verified inhibitor of Eomes. In summary, our results revealed that increased FGL1 expression in HCC affects the proportion and function of CD8^+^ T_RM_ cells by binding to LAG3 on cell membranes, ultimately causing immune escape. These results provide a rationale to consider FGL1 as a new immunotherapeutic target.

## Data availability statement

The original contributions presented in the study are included in the article. Further inquiries can be directed to the corresponding author XC.

## Ethics statement

The studies involving human participants were reviewed and approved by Ethics Committee of Ren Ji Hospital. The patients/participants provided their written informed consent to participate in this study. The animal study was reviewed and approved by Institutional Animal Care and Use Committee at the Shanghai Institute of Materia Medica.

## Author contributions

JZ, and XC designed the research. CY, QQ, YZ, and CW collected samples. CY, QQ, YZ, BH, and RC performed the research. CY, QQ, and QG analyzed data. CY and QQ wrote the manuscript. ZY, LX, JZ, and XC supervised the research and edited the manuscript. All authors contributed to the article and approved the submitted version.
