# Supplementary material for: Fibrinogen-like protein 1 promotes liver-resident memory T-cell exhaustion in hepatocellular carcinoma
Source: Front Immunol. 2023 Mar 13;14:1112672. doi: 10.3389/fimmu.2023.1112672 (PMC10040674; doi:10.3389/fimmu.2023.1112672)
Supplement: Supplementary file 1 [file DataSheet_1.docx]

Supplementary Material

#
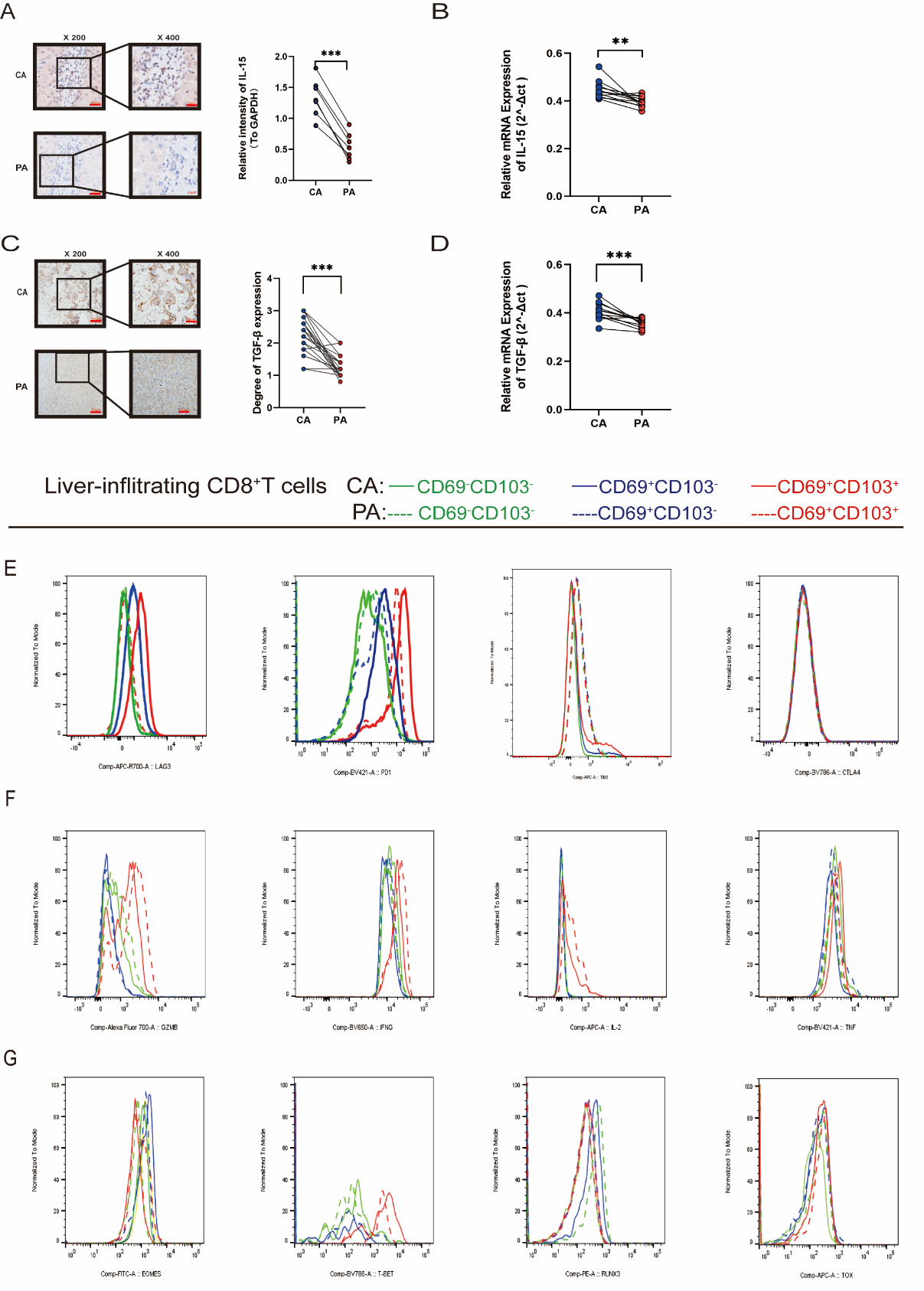
Supplementary Figures

**Figure S1. Intrahepatic microenvironment of HCC patients is conducive to CD103^+^ T cells**

(A) Representative IHC staining of IL-15 (inverted microscope, 200×, 400×) in CA and PA of HCC patients from Cohort 1 (n = 35, nonparametric Mann-Whitney test).

(B) IL-15 mRNA expression levels in hepatocellular CA and PA of patients from Cohort 1 (n = 12, Student’s *t*-test).

(C) Representative IHC staining of TGF-β (inverted microscope, 200×, 400×) in CA and PA of patients from Cohort 1 (n = 35, nonparametric Mann-Whitney test).

(D) TGF-β mRNA expression in CA and precancerous tissues of patients from Cohort 1 (n = 12 Student’s *t*-test).

(**E**) Immune checkpoint expression on CD8^+^ T_RM_ cells and other CD8^+^ T-cell subpopulations in CA and PA of HCC patients from Cohort 1 (n = 20, A paired *t*-test).

(**F**) Cytokine expression in CD8^+^ T_RM_ cells and other CD8^+^ T-cell subpopulations in CA and PA of HCC patients from Cohort 1 (n = 20, A paired *t*-test).

(**G**) Transcription factor expression on CD8^+^ T_RM_ cells and other CD8^+^ T-cell subpopulations in CA and PA of HCC patients from Cohort 1 (n = 20, A paired *t*-test).

(A–G) Bars represent mean ± SEM. CA = carcinoma tissue, HCC = hepatocellular carcinoma, IHC = immunohistochemistry, PA = paracancerous tissue. **P* < 0.01, ****P* < 0.001.


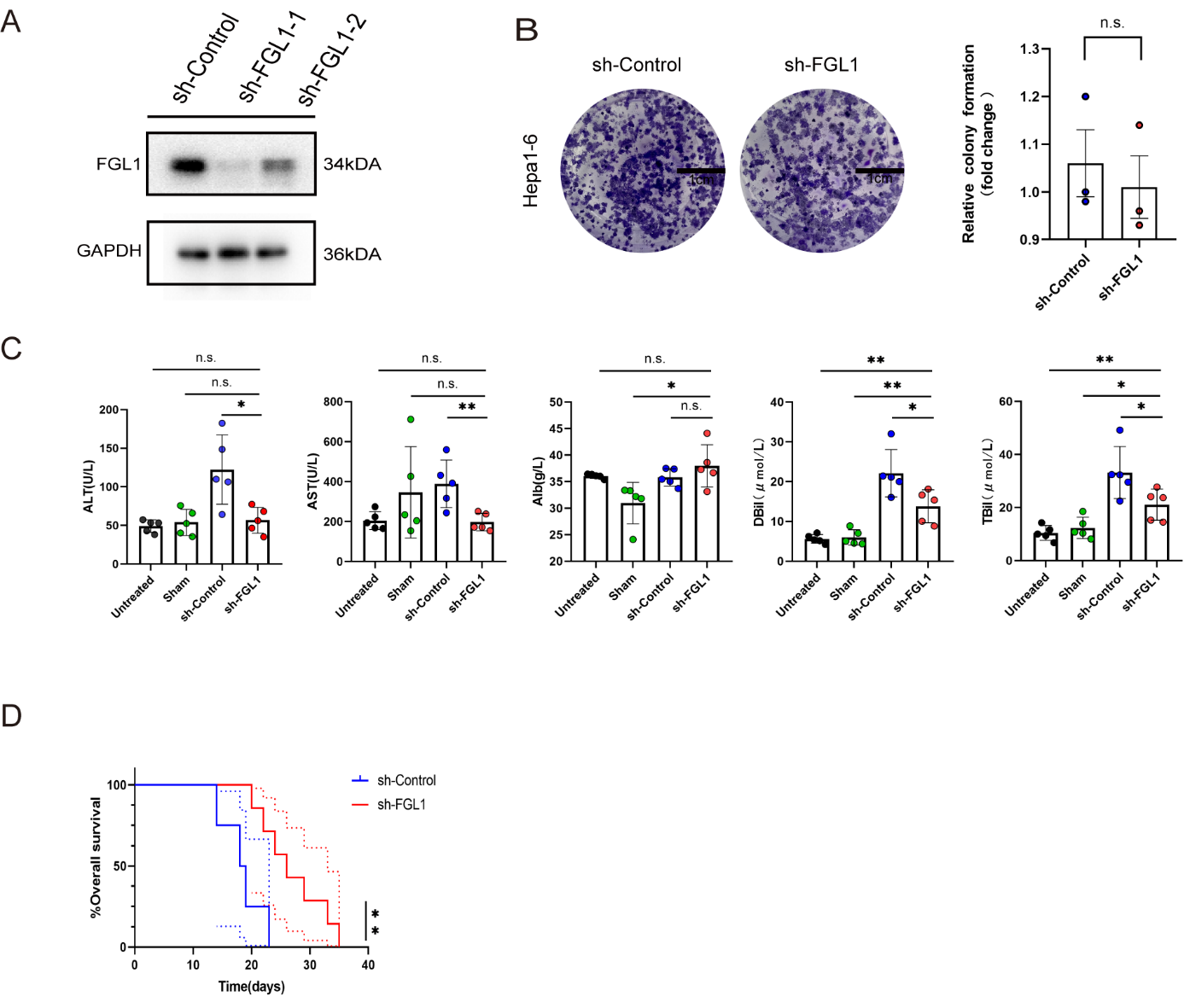


**Figure S2. sh-** ***Fgl1* Hepa1-6 does not significantly alter T_RM_ cell proliferation**

(A) FGL1 protein expression in *Fgl1*-knockdown Hepa1-6 treated with shRNA.

(B) The colony formation ability of *Fgl1-*knockdown Hepa1-6 cells was not significantly affected. (Student’s *t*-test)

(C) Serum ALT, AST, Alb, DBil, and Tbil levels of mice in each group (n = 20, Student’s *t*-test).

(D) Mice treated as above mentioned were further used to estimate the influence of sh-*Fgl1* on overall survival. For this, the survival of mice was assessed daily. Survival curves of the sh-*Fgl1* and sh-Control groups are shown (n=10).

(A–D) Bars represent mean ± SEM. FGL1 = fibrinogen-like protein 1, CA = carcinoma tissue, PA = paracancerous tissue, shRNA = short hairpin RNA. ALT= Alanine aminotransferase, AST= Aspartate aminotransferase, TBil =Total bilirubin, DBil = Direct Bilirubin, Alb=Albumin, **P* < 0.05, ***P* < 0.01.

# Supplementary Tables

## TableS1：Clinical features of HCC patients in cohort 1 of the study

| HCC patients (n=35）^a^ | |
| --- | --- |
| Sex(male/female) | 25/10 |
| Age at surgery (y)^b^ | 57.1±6.4 |
| ALT(U/L) ^b^ | 56.1±23.7 |
| AST(U/L) ^b^ | 77.5±19.1 |
| ALP(U/L) ^b^ | 117.1±26.4 |
| TBil(umol/L) ^b^ | 51.1±15.1 |
| Cirrhosis(yes/no) | 12/23 |
| Tumor size(cm)^b^ | 6.7±4.5 |
| AFP level before resection(mg/L) < 20/20-400/>400 | 14/10/11 |
| a Etiology of liver disease: hepatitis B/C (n =30/2). alcohol-related liver disease (n =2) nonalcoholic steatohepatitis/nonalcoholic fatty liver disease (n=1). b Median ± standard deviation. | |

HCC = hepatocellular carcinoma, ALT= Alanine aminotransferase, AST= Aspartate aminotransferase, ALP= Alkaline phosphatase, TBil =Total bilirubin, AFP= Alpha fetoprotein.

## Table S2： Clinical features of HCC patients in cohort 2 of the study

| HCC patients (n=80）^a^ | |
| --- | --- |
| Sex(male/female) | 69/11 |
| Age at surgery (y)^b^ | 50.5±8.7 |
| ALT(U/L) ^b^ | 359.7±618 |
| AST(U/L) ^b^ | 498．9±917 |
| ALP(U/L) ^b^ | 117.1±26.4 |
| TBil(umol/L) ^b^ | 48.9±55.9 |
| Cirrhosis(yes/no) | 59/21 |
| Tumor size(cm)^b^ | 6.4±4.9 |
| AFP level before resection(mg/L) < 20/20-400/>400 | 21/36/23 |
| a Etiology of liver disease: hepatitis B/C (n =72/0). alcohol-related liver disease (n =7) nonalcoholic steatohepatitis/nonalcoholic fatty liver disease (n=1). b Median ± standard deviation. | |

HCC = hepatocellular carcinoma, ALT= Alanine aminotransferase, AST= Aspartate aminotransferase, ALP= Alkaline phosphatase, TBil =Total bilirubin, AFP= Alpha fetoprotein.

# Antibodies

| **Name** | **Supplier** | **Cat no.** | **Clone no.** |
| --- | --- | --- | --- |
| CD8 | Abcam | ab17147 | C8/144B |
| CD69 | Abcam | ab233396 | EPR21814 |
| CD103 | Abcam | ab224202 | EPR22590-27 |
| IL-15 | Abcam | ab109082 |  |
| TGF-β | Abcam | ab170874 | EPR12078 |
| FGL1 | Abcam | ab275091 | EPR24018 |
| GAPDH | Abcam | ab181602 | EPR16891 |
| BV510/CD3 | BD Biosciences | 564713 | HIT3a |
| PE-CY5/CD8 | BD Biosciences | 561946 | HIT8a |
| BV605/CD8 | BD Biosciences | 564116 | SK1 |
| BUV395/CD4 | BD Biosciences | 564724 | RPA-T4 |
| BB700/CD4 | BD Biosciences | 742157 | OX-35 |
| PE-Cy7/CD69 | BD Biosciences | 557745 | FN50 |
| PE/CD69 | BD Biosciences | 557050 | FN50 |
| APC-R700/CD69 | BD Biosciences | 565155 | FN50 |
| BB515/CD103 | BD Biosciences | 564578 | Ber-ACT8 |
| PerCP-Cy5.5 /CD103 | Biolegend | 350225 | Ber-ACT8 |
| PE/CD103 | BD Biosciences | 550260 | Ber-ACT8 |
| BV421/PD1 | Biolegend | 329920 | EH12.2H7 |
| APC-R700/LAG3 | BD Biosciences | 565775 | T47-530 |
| BV786/CTLA4 | BD Biosciences | 563931 | BNI3 |
| BV785/CTLA4 | Biolegend | 369623 | BNI3 |
| APC/TIM3 | BD Biosciences | 748820 | 7D3 |
| APC/TIM3 | Biolegend | 345011 | F38-2E2 |
| PE/TNF-α | BD Biosciences | 559321 | MAb11 |
| BV421/TNF-α | Biolegend | 502931 | Mab11 |
| BV650/IFN-γ | BD Biosciences | 563416 | 4S. B3 |
| PE/IFN-γ | Biolegend | 506507 | B27 |
| BV421/Granzyme B | Biolegend | 396414 | QA18A28 |
| AF700/ Granzyme B | Biolegend | 372221 | QA16A02 |
| APC/IL-2 | Biolegend | 500310 | MQ1-17H12 |
| EF660/ TOX | eBioscience | 50-6502-80 | TXRX10 |
| PE-Cy7/ Eomes | eBioscience | 25-4875-82 | Dan11mag |
| FITC/ Eomes | BD Biosciences | 11-4877-41 | WD1928 |
| PE/Runx-3 | BD Biosciences | 564814 | R3-5G4 |
| BV786/T-bet | BD Biosciences | 564141 | O4-46 |
| BV650/CD3e | BD Biosciences | 564378 | 145-2C11 |
| BV786/CD8a | BD Biosciences | 563332 | 53-6.7 |
| BV421/CD69 | BD Biosciences | 562920 | H1.2F3 |
| PE/CD103 | BD Biosciences | 557495 | M290 |
| FVS780 | BD Biosciences | 565388 |  |

# Sequence based reagents

| **Name** | **Sequence** | **Supplier** |
| --- | --- | --- |
| Primer of IL-15 | forward, 5′-AACAGAAGCCAACTGGGTGAATG -3′, reverse, 5′-CTCCAAGAGAAAGCACTTCATTGC -3′ | Tsingke Biotechnology Co |
| Primer of TGF-β | forward ，5′- TACCTGAACCCGTGTTGCTCTC -3′, reverse,5′-GTTGCTGAGGTATCGCCAGGAA -3′ | Tsingke Biotechnology Co |
| Primer of GAPDH | forward ，5′-ATGTTCCAGTATGA CTCCACTCAC -3′, reverse 5′-GACACAGTAG ACTCCACGACATA -3′ | Tsingke Biotechnology Co |
| ShRNA targeting *Fgl1* | 5′- GTATGCAGATTGTTCAGAGAT -3′, 5′-CCATTGCTCTGATGATGGGAA -3′ | Shanghai Zorin Biotechnology |

# Software

| **Software name** | **Manufacturer** | **Version** |
| --- | --- | --- |
| Image J | National Institutes of Health | 1.46 |
| FlowJo software | Tree star | 10.6.2 |
| Prism | GraphPad Software Inc | 8.0 |

# Other (e.g. drugs, proteins, vectors etc.)

| **Name** | **Supplier** | **Cat no.** |
| --- | --- | --- |
| Recombinant human IL-2 | Peprotech | 200-02 |
| Recombinant human IL-15 | R&D systems | BT-015-010 |
| Recombinant human TGF-β | Peprotech | 100-21 |
| DMEM | Thermo Fisher Scientific | 11965092 |
| RPMI 1640 | Hyclone | SH30809 |
| Penicillin–streptomycin | Thermo Fisher Scientific | M7145 |
| FBS | Gibco | 12662029 |
| Ficoll-Paque Plus | GE Life | 17144002 |
| Percoll | GE Life | 17089102 |
| LSR Fortessa flow cytometer | BD Biosciences |  |
| LSR Fortessa X-20 flow cytometer | BD Biosciences |  |
